# Identification of immune and major depressive disorder-related diagnostic markers for early nonalcoholic fatty liver disease by WGCNA and machine learning

**DOI:** 10.3389/fbinf.2025.1594971

**Published:** 2025-06-26

**Authors:** Yuyun Jia, Yanping Cao, Qin Yin, Xueqian Li, Xiu Wen

**Affiliations:** Department of Gastroenterology, Nanjing Drum Tower Hospital, Affiliated Hospital of Medical School, Nanjing University, Nanjing, China

**Keywords:** major depressive disorder, nonalcoholic fatty liver disease, simple steatosis, nonalcoholic steatohepatitis, machine learning, weighted gene co-expression network analysis, psychological nursing intervention

## Abstract

**Background:**

Major depressive disorder (MDD) and nonalcoholic fatty liver disease (NAFLD) are highly prevalent conditions that exhibit significant pathophysiological overlap, particularly in metabolic and immune pathways.

**Objective:**

This study aims to bridge this gap by integrating transcriptomic data from publicly available repositories and advanced machine learning algorithms to identify novel biomarkers and construct a predictive model facilitates the provision of clinical psychological nursing interventions for early-stage NAFLD in MDD patients.

**Method:**

We systematically analyzed transcriptomic data of simple steatosis (SS), nonalcoholic steatohepatitis (NASH), and major depressive disorder (MDD) from GEO databases to construct and validate a diagnostic model. After removing batch effects, we identified differentially expressed genes (DEGs) that distinguished disease and control groups. We further applied Weighted Gene Co-expression Network Analysis (WGCNA) to identify immune-related genes in SS/NASH patients versus controls. The intersection of shared DEGs across both conditions and WGCNA-identified genes was determined and subjected to functional enrichment analysis. Immune cell infiltration levels were quantified using single-sample gene set enrichment analysis (ssGSEA). A predictive model for SS/NASH was developed by evaluating nine machine-learning algorithms with 10-fold cross-validation on the datasets.

**Results:**

Fourteen genes strongly linked to both the immune system and the two conditions were identified. Immune cell infiltration profiling revealed distinct immune landscapes in patients versus healthy controls. Moreover, an eight-gene signature was developed, demonstrating superior diagnostic accuracy in both testing and training cohorts. Notably, these eight genes were found to correlate with the severity of early-stage NAFLD.

**Conclusion:**

This study established a predictive model for early-stage NAFLD through the integration of bioinformatics and machine learning approaches, with a focus on immune- and MDD-related genes. The eight-gene signature identified in this study represents a novel diagnostic tool for precision medicine, enabling targeted psychological nursing intervention in comorbid populations.

## Introduction

Major depressive disorder (MDD) is a complex disorder characterized by multiple impairments, with emotional dysfunction being the primary one (Wang et al., 2025). MDD is intricately linked to nonalcoholic fatty liver disease (NAFLD) through shared pathophysiological pathways, including immune-inflammatory dysregulation, metabolic dysfunction, and neuroendocrine imbalance (Zhang et al., 2024). NAFLD has emerged as a major global public health challenge, affecting over one-quarter of the world’s population (Nie et al., 2025). Recent investigations have underscored the critical need to characterize the bidirectional relationship between neuropsychiatric disorders and NAFLD, as this mechanistic understanding is essential to mitigate the progression of comorbid metabolic and psychiatric pathologies (Wang S. et al., 2024; Xu et al., 2024). Accumulating evidence demonstrates that MDD predisposes individuals to NAFLD through stress-mediated dysregulation of neuroendocrine and inflammatory axes (Shao et al., 2021).

NAFLD represents a histopathological spectrum of liver disorders, ranging from simple steatosis (SS) to nonalcoholic steatohepatitis (NASH), with NASH potentially progressing to cirrhosis and hepatocellular carcinoma when untreated (Figge et al., 2021). SS is defined as triglyceride accumulation in hepatocytes without histological evidence of hepatocellular injury (De and Duseja, 2020). In contrast, NASH is characterized by lobular inflammation, hepatocellular ballooning, and progressive fibrosis, which may evolve into cirrhosis and end-stage liver disease (Xu et al., 2025).

Recent advancements in genomics and bioinformatics have shed new light on the molecular mechanisms underpinning MDD and NAFLD. A study by Arold et al. identified shared genetic pathways governing immune regulation and metabolic dysfunction across these disorders (Arold et al., 2024). Another study revealed that specific gene expression profiles linked to the pathogenesis of MDD and NAFLD exhibit a strong association with the next-generation epigenetic aging clock, CheekAge (Shokh et al., 2025). These findings underscore the potential of utilizing genetic markers to develop predictive models for the early diagnosis of NAFLD in individuals with MDD.

Despite the accumulating evidence linking MDD and NAFLD, substantial gaps persist in research investigating the mechanistic link between MDD and early-stage NAFLD (Zhu et al., 2020), specifically SS (Mazzolini et al., 2020) and NASH (Xu et al., 2025). This evidentiary gap is particularly problematic, as early detection and intervention are paramount for averting disease progression and ameliorating patient outcomes. Consequently, an urgent imperative exists to develop diagnostic tools for identifying early-stage NAFLD in individuals with MDD, thereby enabling timely therapeutic interventions.

To address this critical gap, we propose an integrated machine-learning framework for developing a diagnostic model of early-stage NAFLD using MDD-related genes. By integrating publicly available transcriptomic data from the Gene Expression Omnibus (GEO) database, we sought to identify differentially expressed genes (DEGs) that distinguish simple steatosis (SS) or nonalcoholic steatohepatitis (NASH) from healthy controls. We further applied Weighted Gene Co-expression Network Analysis (WGCNA) to isolate immune-related gene modules. Functional enrichment analyses of immune- and MDD-associated genes were performed to characterize key biological pathways, while single-sample gene set enrichment analysis (ssGSEA) was used to quantify immune cell infiltration levels, providing a comprehensive immune landscape for both conditions.

## Materials and methods

### Data collection and batch effect removal

The search terms “simple steatosis (SS)”, “nonalcoholic steatohepatitis (NASH) ”, or “major depressive disorder (MDD)” were used to retrieve datasets from the NCBI Gene Expression Omnibus (GEO; https://www.ncbi.nlm.nih.gov/geo/).

Specifically, we selected two datasets (GSE76826 (Miyata et al., 2016) and GSE98793 (Leday et al., 2018)) that included samples from patients with MDD and healthy controls. Additionally, owing to the limited sample size of the SS and NASH cohorts, we merged four datasets (GSE48452 (Ahrens et al., 2013), GSE63067 (Frades et al., 2015), GSE126848 (Suppli et al., 2019), and GSE89632 (Pettinelli et al., 2022)) that included patients with SS, NASH, and healthy controls.

As the data were sourced from multiple studies, batch effects may have confounded the results. To mitigate this issue, we employed the “ComBat” algorithm (Bostami et al., 2022), which is widely used for batch correction in genomic studies (Leek et al., 2012). This approach mitigates systematic biases arising from divergent experimental conditions, thereby ensuring that downstream analyses remain uncompromised by technical artifacts.

### Identification of differentially expressed genes (DEGs)

Differentially expressed genes (DEGs) were identified using the limma (3.60.6) package in R (version 4.4.2), a tool specialized for RNA-seq data analysis. This package compares gene expression profiles across disease and control groups to identify significantly upregulated or downregulated genes. For each dataset, significance thresholds were set at |log_2_fold change (FC)| >0.25 and false discovery rate (FDR) <0.05. Following DEG identification in MDD, SS, and NASH datasets, overlap analysis was performed to characterize commonly dysregulated genes, which are hypothesized to underlie the shared pathophysiology of these conditions.

### Construction of weighted gene co-expression network analysis (WGCNA)

To perform Weighted Gene Co-expression Network Analysis (WGCNA) on combined datasets (GSE48452, GSE63067, GSE126848, and GSE89632), the pickSoftThreshold function from the WGCNA R package was employed to determine optimal gene co-expression thresholds. Filtering involved selecting the top 5,000 genes with the highest absolute median differences to prioritize transcriptionally variable genes. During preprocessing, genes with missing values or zero variance were excluded to maintain analytical rigor.

Scale-free network topology criteria (requiring a scale-free fit index R^2^ ≥ 0.85) guided adjacency matrix construction, which was subsequently transformed into a Topological Overlap Matrix (TOM). Genes with similar expression profiles were grouped via hierarchical clustering with average linkage. Key gene modules were detected using selection criteria: a minimum module size of 30 genes and a cut height of 0.25. Finally, Pearson’s correlation coefficients between gene modules and disease-related target traits were calculated to identify significant associations.

### Enrichment analysis of shared immune and MDD-related genes

To characterize the biological functions of identified shared DEGs, we performed Gene Ontology (GO), Kyoto Encyclopedia of Genes and Genomes (KEGG) (Kanehisa and Goto, 2000) pathway enrichment, and Disease Ontology Semantic and Enrichment (DOSE) analyses. These analyses utilized the ggplot2 (version 3.5.2), enrichplot (version 1.24.4), clusterProfiler (version 4.12.6), and org. Hs.e.g.,.db (version 3.19.1) R packages.

To ensure robust statistical inference in multiple hypothesis testing, we employed the Benjamini-Hochberg (BH) procedure to control the false discovery rate (FDR), which generates q-values estimating the proportion of false positives among significant results. Following computation of raw p-values, the BH procedure sorts and applies a formula to derive q-values. When q-values ≥0.05 (failing FDR control), corresponding raw p-values are reported. This strategy balances strict FDR control with unadjusted evidence, enabling evaluation across stringency levels. By prioritizing q-values for FDR-controlled findings and including p-values for non-significant results, we present a comprehensive view of statistical evidence in multiple testing contexts.

### Immune-related pathways in each condition

Building on the established role of immune pathways, we prioritized the characterization of immune-related signaling networks. Specifically, we investigated pathways governing inflammation, immune cell activation, and cytokine signaling. Immune cell infiltration levels were quantified using single-sample gene set enrichment analysis (ssGSEA) (Gong et al., 2024), a method that computes enrichment scores for distinct immune cell subsets based on gene expression profiles. We then compared immune cell infiltration profiles between patient and healthy control groups.

### Machine learning algorithms

To develop a predictive model for diagnosing simple steatosis (SS) or nonalcoholic steatohepatitis (NASH) in individuals with major depressive disorder (MDD), we evaluated nine machine learning algorithms: Generalized Boosted Regression Modeling (GBM), Linear Discriminant Analysis (LDA), Elastic Net (Enet), Support Vector Machine (SVM), Ridge Regression, Naive Bayes, StepGLM, generalized linear model boosting (glmBoost), and eXtreme Gradient Boosting (XGBoost).

First, the initial data were preprocessed. This entailed removing missing values and outliers, followed by Z-score standardization. Using this method, each feature was adjusted to have a mean of 0 and standard deviation of 1, thereby mitigating the effect of feature scale disparities.

Subsequently, the dataset was randomly partitioned into training (70%) and testing (30%) subsets. During model training, we utilized six machine learning algorithms: Elastic Net regression (λ = 0.1), Ridge regression (λ = 1.0), Support Vector Machine (SVM, C = 1.0, γ = 0.01), Linear Discriminant Analysis (LDA), Gradient Boosting Machine (GBM, learning rate = 0.1, 100 trees), and eXtreme Gradient Boosting (XGBoost, learning rate = 0.01, 150 trees). Models were trained on the training set, with hyperparameters optimized via cross-validation.

For model evaluation, we calculated the area under the curve (AUC) at a threshold of 0.7 using the testing set to assess classification performance. AUC values were computed using the RunEval function, and model performance was visualized via a heatmap generated with the SimpleHeatmap function. The model with the highest AUC was selected as the optimal classifier. Calibration curves were generated to evaluate diagnostic model accuracy.

### Statistical analysis

Statistical analyses were conducted using R (version 4.4.2). For two-group comparisons, Student’s t-test was used for continuous variables, and Pearson’s chi-squared test for categorical data. One-way analysis of variance (ANOVA) was applied for multiple group comparisons of continuous variables, followed by the Benjamini-Hochberg (BH) procedure to control the false discovery rate (FDR) for multiple testing correction. Statistical significance was defined as p < 0.05.

## Results

### Remove the batch effects

**FIGURE 1:**
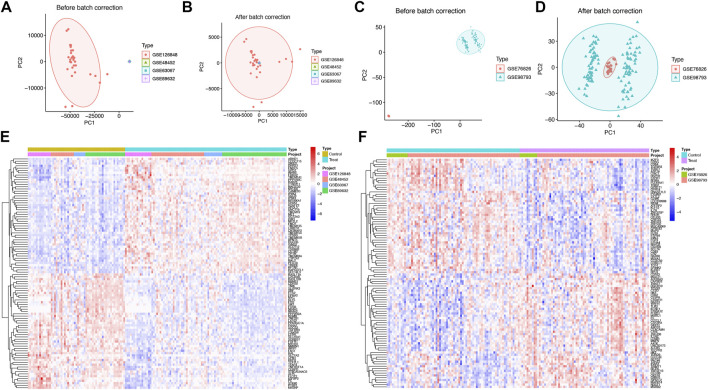
Integration of SS/NASH and MDD datasets with transcriptional changes. **(A,B)** Principal component analysis (PCA) of 4 SS/NASH datasets (GSE48452, GSE63067, GSE126848, GSE89632) before **(A)** and after **(B)** batch correction. **(C,D)** PCA of two MDD datasets (GSE76826, GSE98793) before **(C)** and after **(D)** batch correction. **(E)** Heatmap of differentially expressed genes (DEGs) in SS/NASH vs. controls. **(F)** Heatmap of DEGs in MDD vs. controls. DEGs, differentially expressed genes.

Raw transcriptomic data for simple steatosis (SS), nonalcoholic steatohepatitis (NASH), and control samples were obtained from the GEO database. Following batch effect removal, data were integrated and normalized, yielding a processed cohort comprising 98 SS/NASH patients and 59 healthy controls (Figures 1A,B; Table 1). Similarly, raw datasets of major depressive disorder (MDD) and control groups were combined after batch effect correction (Figures 1C,D), yielding a normalized validation cohort with 138 MDD patients and 76 healthy controls (Table 1). Consequentially, batch effects were significantly mitigated post-correction.

**TABLE 1:** Basic information of GEO datasets used in the study.

GSE series	Disease^a^	Samples	Platform
GSE48452	SS or NASH	14 SS, 18 NASH, and 14 health control	GPL11532
GSE63067	SS or NASH	2 SS, 9 NASH, and 7 health control	GPL570
GSE126848	NASH	16 NASH and 14 health control	GPL18573
GSE89632	SS or NASH	20 SS, 19 NASH and 24 health control	GPL14951
GSE76826	MDD	10 MDD and 12 health control	GPL17077
GSE98793	MDD	128 MDD and 64 health control	GPL570

^a^ SS, simple steatosis; NASH, nonalcoholic steatohepatitis; MDD, major depressive disorder.

### Identifying of DEGs

Given the crosstalk between SS/NASH and MDD, we performed limma analysis on these cohorts to identify MDD-associated differentially expressed genes (DEGs) linked to SS/NASH. A total of 2,606 DEGs were identified in the SS/NASH cohorts (Figure 1E), including 1,507 upregulated and 1,099 downregulated genes. In the MDD cohort, 209 DEGs were detected (Figure 1F), with 99 upregulated and 110 downregulated genes.

### Construction of weighted gene co-expression networks

**FIGURE 2:**
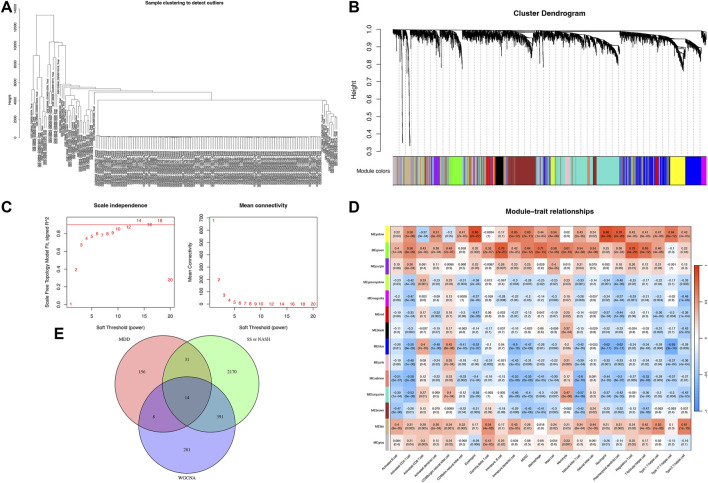
Weighted gene co-expression network analysis (WGCNA) for SS/NASH. **(A)** Sample clustering based on expression levels after batch correction. Tree branches represent individual samples, with no outliers identified. **(B)** Module formation and merging processes below the clustering tree. **(C)** Determination of the optimal soft-threshold power for sample data. **(D)** Heatmap showing correlations between module eigengenes and immune cell infiltration profiles. **(E)** Venn diagrams depicting intersecting genes from SS, NASH, MDD cohorts, and WGCNA modules. WGCNA, weighted gene co-expression network analysis.

Weighted gene co-expression network analysis (WGCNA) was employed to explore the relationship between immune cell composition and gene expression in SS and NASH datasets. Following batch effect correction, unsupervised clustering was performed to classify patients based on gene expression profiles in SS/NASH samples (Figures 2A,B). An optimal soft threshold of 16 was determined for the dataset, achieving a scale-free fit index (R^2^ = 0.85; Figure 2C), and 14 gene modules were identified (Figure 2D).

Specifically, the yellow and green modules exhibited significant positive correlations with activated CD4^+^ T cells, activated dendritic cells, eosinophils, immature dendritic cells, myeloid-derived suppressor cells (MDSCs), regulatory T cells, and follicular helper T cells. The purple module was strongly associated with activated CD4^+^ T cells, eosinophils, immature B cells, immature dendritic cells, MDSCs, mast cells, and T helper 1 (Th1) cells. The tan module demonstrated robust positive correlations with activated B cells, activated CD4^+^ T cells, activated CD8^+^ T cells, activated dendritic cells, CD56 bright natural killer cells, CD56 dim natural killer cells, gamma delta T cells, immature dendritic cells, natural killer T cells, natural killer cells, T follicular helper cells, and T helper 1/2 (Th1/Th2) cells (Figure 2D). Given their critical association with infiltrating immune cells, the yellow, green, purple, and tan modules were prioritized for downstream analysis.

Subsequently, an intersection analysis was performed between DEGs from SS, NASH, and MDD cohorts and genes within these modules. This analysis identified 14 shared genes, which were selected for further functional characterization (Figure 2E).

### Functional enrichment of the shared genes

**FIGURE 3:**
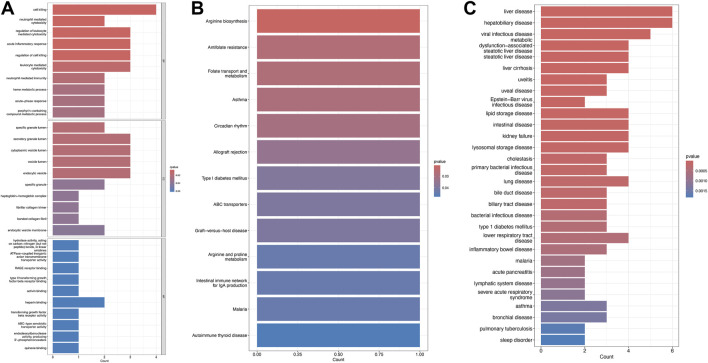
Enrichment analysis of shared DEGs. **(A)** Gene Ontology (GO) enrichment for biological processes (BP), cellular components (CC), and molecular functions (MF). **(B)** Kyoto Encyclopedia of Genes and Genomes (KEGG) pathway enrichment. **(C)** Disease Ontology enrichment, with terms color-coded by significance.

Gene Ontology (GO) enrichment analysis identified overrepresented biological processes, including cell-mediated cytotoxicity, neutrophil-mediated cytotoxicity, regulation of leukocyte-mediated cytotoxicity, and acute inflammatory responses. Enriched cellular components included secretory granule lumen, cytoplasmic vesicle lumen, vesicle lumen, and endocytic vesicles, while heparin binding was the prominent molecular function (Figure 3A). Kyoto Encyclopedia of Genes and Genomes (KEGG) pathway analysis further revealed significant enrichment in arginine biosynthesis, antifolate resistance, folate transport and metabolism, asthma, and circadian rhythm pathways (Figure 3B).

Disease Ontology Semantic and Enrichment (DOSE) analysis demonstrated that shared genes were significantly associated with metabolic dysfunction–associated steatotic liver disease, steatotic liver disease, liver cirrhosis, lipid storage disease, liver disease, lysosomal storage disease, viral infectious disease, and hepatobiliary disease (Figure 3C).

### Immune cell infiltration analysis of both conditions

**FIGURE 4:**
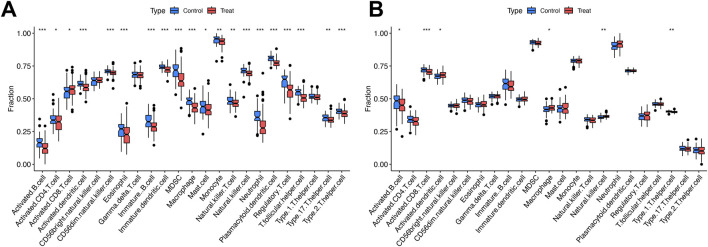
Immune cell abundance comparisons between disease groups and controls. **(A)** Boxplots of immune cell proportions in SS/NASH vs. controls. **(B)** Boxplots of immune cell proportions in MDD vs. controls. Significance: *P < 0.05, **P < 0.01, ***P < 0.001.

Figure 4A revealed elevated proportions of activated CD8^+^ T cells in simple steatosis (SS) and nonalcoholic steatohepatitis (NASH) cohorts. By contrast, the abundance of activated B cells, activated CD4^+^ T cells, activated dendritic cells, CD56 dim natural killer cells, eosinophils, immature B cells, immature dendritic cells, myeloid-derived suppressor cells (MDSCs), macrophages, natural killer cells, neutrophils, plasmacytoid dendritic cells, regulatory T cells, T follicular helper cells, T helper 17 (Th17) cells, and T helper 2 (Th2) cells was reduced relative to controls (Figure 4A).

Figure 4B showed increased proportions of activated dendritic cells, macrophages, and natural killer cells in the major depressive disorder (MDD) cohort. Conversely, activated B cells, activated CD8^+^ T cells, and T helper 1 (Th1) cells exhibited reduced abundance compared with controls (Figure 4B).

### Developing a diagnostic model for MDD-related early NAFLD via machine learning

**FIGURE 5:**
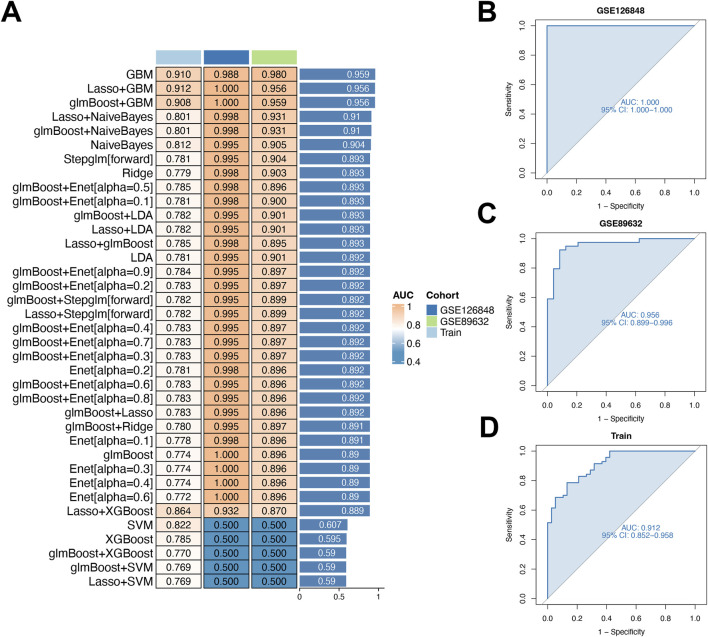
Diagnostic performance of the model for early-stage NAFLD in MDD patients. **(A)** Evaluation of nine machine-learning algorithm combinations via 10-fold cross-validation. **(B–D)** Receiver operating characteristic (ROC) curves for two validation cohorts and the training cohort.

Using 10-fold cross-validation, we evaluated 12 machine learning algorithms to develop a diagnostic model using shared genes. This analysis, performed on integrated datasets (GSE48452, GSE63067, GSE126848, GSE89632), aimed to identify the most reliable model (Figures 5A–D). The LASSO and GBM algorithms were employed to build the final model, which demonstrated optimal performance. These algorithms identified eight key genes (TGFBR3, S100A12, TP53I3, RASGEF1B, NFIL3, CD163, KLRB1, COL5A3) and facilitated selection of the most robust diagnostic model.

### Assessment of our model

**FIGURE 6:**
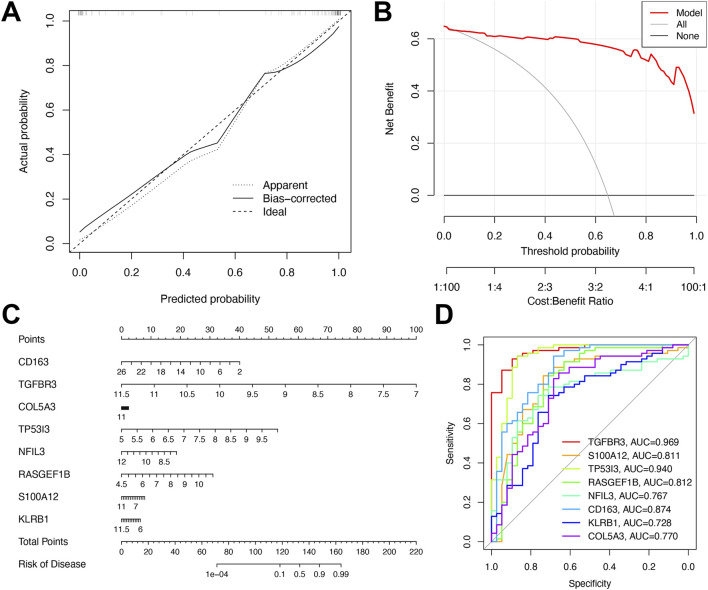
Performance assessment of the diagnostic model. **(A)** Calibration plot showing agreement between predicted and observed probabilities. **(B)** Decision curve analysis of the 8-gene model for MDD-related early-stage NAFLD. **(C)** Nomogram for predicting MDD-related early-stage NAFLD based on shared DEGs. **(D)** ROC curves of the 8-gene signature for early-stage NAFLD diagnosis.

As depicted in Figure 6A, the calibration curves of the diagnostic model closely mirrored the ideal diagonal line in both cohorts, demonstrating strong consistency between predicted probabilities and observed clinical outcomes. This indicated excellent calibration performance. The clinical utility of the model was further validated by decision curve analysis (Figure 6B), which showed that the nomogram yielded the highest net benefit across a broad range of threshold probabilities. Following multivariate analysis, nomograms integrating the eight-gene signature were developed to predict early NAFLD risk (Figure 6C). ROC curve analysis confirmed the superior diagnostic efficacy of the eight-gene signature, with TGFBR3 and TP53I3 exhibiting AUC values (0.940–0.969) that significantly outperformed other genes (Figure 6D).

### Clinical relevance and gene expression heatmap analysis

**FIGURE 7:**
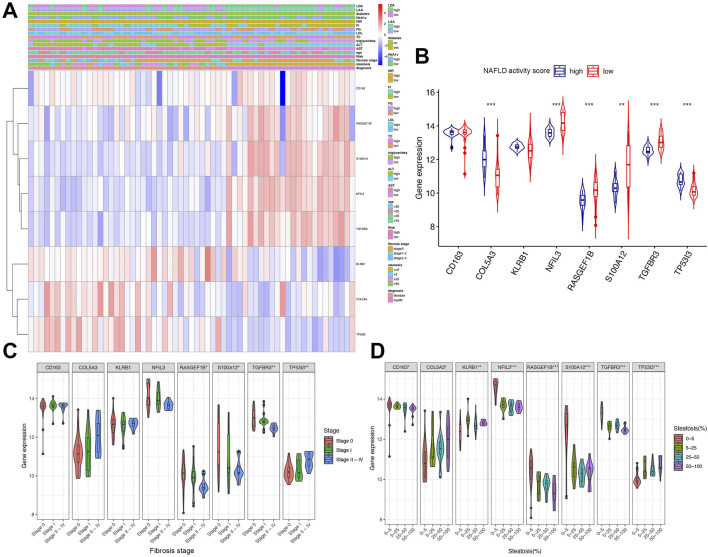
Clinical relevance and gene expression heatmap analysis. **(A)** Heatmap of gene expression correlated with clinical parameters (risk score based on NAFLD activity score; score ≥4 defined as high risk). **(B)** Expression of eight key genes in high vs. low NAFLD activity score groups. **(C,D)** Expression of gene signatures across fibrosis stages and steatosis degrees. AST, aspartate transaminase (U/L); ALT, alanine transaminase (U/L); TC, total cholesterol (mmol/L); LDL, low-density lipoprotein (mmol/L); FG, fasting glucose (mmol/L); FI, fasting insulin (pmol/L); HIR, homeostatic insulin resistance; HbA1c, hemoglobin A1c; LAA, liver arachidonic acid (% of total lipids); LDA, liver docosahexaenoic acid (% of total lipids). Groups for continuous variables were divided by median. Significance: *P < 0.05, **P < 0.01, ***P < 0.001.

A heatmap was generated to visualize the correlation between the expression levels of eight immune-related genes and clinical parameters in early NAFLD patients using the GSE89632 dataset. Genes including KLRB1, COL5A3, and TP53I3 exhibited higher expression in groups with elevated Aspartate transaminase (AST), Alanine transaminase (ALT), Triglycerides, Fasting glucose (FG), Homeostatic insulin resistance (HIR), and Hemoglobin A1c (HbA1c). Steatosis severity was associated with upregulation of COL5A3 and TP53I3, whereas upregulated RASGEF1B, S100A12, and TGFBR3 were linked to early-stage liver steatosis (Figure 7A).

Figure 7B illustrated the expression of these eight genes across different NAFLD activity score groups. Compared with the low-score group, COL5A3 and TP53I3 showed significant upregulation, while NFIL3, RASGEF1B, S100A12, and TGFBR3 were significantly downregulated (Figure 7B). Figure 7C depicted gene expression across fibrosis stages, revealing significant variation in RASGEF1B, S100A12, TGFBR3, and TP53I3 expression (Figure 7C). Figure 7D demonstrated that all identified genes exhibited significant expression differences corresponding to varying degrees of steatosis (Figure 7D).

## Discussion

This study sought to develop an integrated machine-learning framework for the early diagnosis of nonalcoholic fatty liver disease (NAFLD) in individuals with major depressive disorder (MDD). By integrating transcriptomic data with advanced computational approaches, we identified an eight-gene signature that exhibited robust diagnostic accuracy for early-stage NAFLD (simple steatosis [SS]/nonalcoholic steatohepatitis [NASH]) in MDD patients. The model demonstrated high sensitivity and specificity in both training and validation cohorts, underscoring its translational potential for clinical application. Furthermore, we identified putative therapeutic compounds targeting these genes, which warrant further investigation in future therapeutic development.

Health conditions and socioeconomic status mediate the causal effect of reproductive traits on NAFLD (Wang Q. et al., 2024). Other studies have also investigated the relationship between MDD and NAFLD, highlighting the shared genetic and metabolic pathways underlying these conditions (Li et al., 2024). Chen et al. elucidated the mechanisms underlying the comorbidity of MDD and multiple gastrointestinal disorders (Chen et al., 2023). Previous studies have also reported significant overlaps in gene expression profiles between MDD and NAFLD, particularly in the pathways related to immune regulation and lipid metabolism (Shao et al., 2021). Similarly, Zhou et al. revealed a prospective association between depression and severe NAFLD, thus potentially necessitating clinical monitoring of individuals with depression for severe NAFLD (Zhou et al., 2024). These findings align with our results, as we also observed significant enrichment of immune-related pathways (Sales et al., 2023) and metabolic dysfunction (Zhu et al., 2023) in our shared DEGs (Le et al., 2021). However, unlike previous studies that primarily focused on the later stages of NAFLD, our study specifically targeted patients with early-stage NAFLD.

The identification of 14 shared differentially expressed genes (DEGs) between major depressive disorder (MDD) and early nonalcoholic fatty liver disease (NAFLD; including SS and NASH) highlights their putative role in immune-mediated pathways. Functional enrichment analysis revealed significant overrepresentation of acute inflammatory response and cell-mediated cytotoxicity pathways, consistent with the immune cell infiltration profiles observed in both conditions. In this study, SS and NASH cohorts exhibited elevated activated CD8^+^ T cells, whereas the MDD cohort showed increased activated dendritic cells, macrophages, and natural killer cells—key mediators of proinflammatory responses (Sun et al., 2025). These shared genes may mediate crosstalk between the neuroinflammatory mechanisms of MDD and hepatic immune surveillance in NAFLD, where chronic stress-induced glucocorticoid elevation (a known driver of MDD) (Lukic et al., 2015) disrupts hepatic immune cell homeostasis, thereby promoting lipid accumulation and hepatocyte damage.

KEGG pathway analysis of the shared DEGs highlighted enrichment in antifolate resistance, folate metabolism, and circadian rhythms linked to both psychiatric and metabolic disorders. For example, folate dysmetabolism is associated with MDD (Carboni et al., 2021) and NAFLD (Jung et al., 2025). Disease ontology analysis further implicated these genes in metabolic dysfunction–associated steatotic liver disease and cirrhosis, highlighting their role in disease progression. Notably, the tan module—strongly correlated with activated B cells and natural killer T cells—may represent a key interface where adaptive immune responses drive hepatic inflammation in NAFLD and neuroinflammation in MDD. This hypothesis was supported by recent evidence of gut–liver–brain axis interactions in comorbid conditions, which underscores the module’s potential role in bridging immune responses across hepatic and neurological contexts (Li et al., 2025; Fan et al., 2025).

The overlap of DEGs in immune cell infiltration and metabolic pathways suggests that targeting these shared genes may offer dual benefits in MDD and early NAFLD. For example, heparin-binding proteins (a highlighted molecular function) are involved in immune cell trafficking and may represent novel therapeutic targets for mitigate both neuroinflammation (Maurya et al., 2016) and hepatic steatosis (Goikoetxea-Usandizaga et al., 2022). Additionally, the identified gene modules (yellow, green, purple, and tan) provide a framework for developing multi-omics biomarkers to predict disease progression in patients with comorbid MDD and NAFLD. Given the rising global prevalence of both conditions, these findings underscore the need for integrated approaches that address immune-metabolic dysregulation, and potentially improve early nursing intervention strategies and clinical outcomes (Arold et al., 2024).

Studies have also found that nearly one in six patients with cirrhosis has moderately severe to severe depression, and nearly half of them have moderate to severe anxiety (Hernaez et al., 2022). Therefore, early detection during the asymptomatic phase offers a critical therapeutic window for interrupting disease progression and mitigating subsequent psychiatric comorbidities (Zimbrean and Jakab, 2025). This distinction is crucial because early detection can significantly affect patient outcomes by enabling timely interventions (Wigg et al., 2025). The consistency in the predictive accuracy suggests that our model is not overfitted and can be effectively applied to new, unseen data. The calibration curves of our diagnostic model aligned closely with the perfectly calibrated diagonal in both the validation sets (Demir et al., 2024). This near-ideal overlap demonstrates robust concordance between the predicted probabilities and observed clinical outcomes, underscoring the exceptional calibration accuracy of the model.

CD163, a hemoglobin scavenger receptor, is a macrophage-specific protein associated with the “alternative activation” (M2) phenotype that plays a pivotal role in dampening inflammatory responses. Recent studies have established midbrain CD163+ macrophages as key players in MDD pathophysiology, opening new avenues for developing anti-inflammatory approaches that synergize with conventional antidepressants to enhance therapeutic efficacy (Mendez-Victoriano et al., 2024). Other studies have also demonstrated that CD163 is a pivotal mediator of microglial hypoactivity in MDD, opening avenues for developing CD163-targeted therapies that restore microglial effector function (Scheepstra et al., 2023). Future research should prioritize clinical trials evaluating CD163-inducing agents and explore their synergistic effects with conventional antidepressants to improve the treatment efficacy for MDD. KLRB1 (killer cell lectin-like receptor subfamily B member 1) is another critical mediator linking immune-inflammatory pathways to MDD pathogenesis (Zhao et al., 2021). Mechanistically, KLRB1 may influence MDD through two plausible pathways: (1) modulating microglial activation and neuroinflammation, as KLRB1+ immune cells have been shown to infiltrate the brain during chronic stress, promoting proinflammatory cytokine release (Winans et al., 2023); and (2) interfering with the hypothalamic-pituitary-adrenal (HPA) axis (Wei and Hong, 2024), given KLRB1’s role in stress-induced immune dysregulation. The association between KLRB1 and inflammatory markers in patients with MDD further supports its role in bridging the gap between immunity and depression.

The significance of our model lies in its ability to facilitate early detection of NAFLD in patients with MDD. Early intervention is critical in managing NAFLD, as it can prevent the progression to more severe forms of liver disease, such as cirrhosis (Ang et al., 2025) and hepatocellular carcinoma (Moon et al., 2025). By identifying at-risk individuals using a reliable diagnostic tool, healthcare providers can implement personalized treatment plans that address both MDD and NAFLD, thereby improving the overall patient outcomes. This study also offers valuable insights that could inform the development of comprehensive intervention strategies for managing depression in clinical settings (Jiang et al., 2024). By identifying at-risk individuals using a reliable diagnostic tool, healthcare providers can implement personalized treatment plans that address both MDD and early stage NAFLD (Nurcahyanti et al., 2022). Additionally, the identification of therapeutic targets based on our gene signature opens new avenues for drug development, potentially leading to more effective treatments for this comorbid condition (Geng et al., 2025).

## Limitations

While our study offers valuable insights into early NAFLD diagnosis in MDD patients, it has notable limitations. A primary constraint is the limited number of shared DEGs. The identification of only a small number of intersecting DEGs between MDD and early NAFLD (simple steatosis/nonalcoholic steatohepatitis, SS/NASH) significantly impacted model development, potentially restricting the robustness of our diagnostic framework (Suarez-Barcena et al., 2025). Due to the paucity of overlapping genes, numerous machine-learning algorithms were excluded during the initial evaluation phase. Specifically, complex models require a substantial number of input features to achieve optimal performance (Su et al., 2025).

With a reduced feature space, these models struggled to capture intricate data relationships, leading to suboptimal performance. Consequently, we relied on simpler models, which are less sensitive to input dimensionality but may lack the predictive power of complex algorithms. The relatively small sample size of the external validation dataset may limit the generalizability of findings to broader populations. Although rigorous feature selection and 10-fold cross-validation mitigated overfitting, limited samples still constrained the complexity of reliably trainable models.

Future studies should prioritize large, multi-cohort validation and advanced feature-reduction strategies to optimize model robustness. Notably, the identified gene signature presents dual utility in precision medicine: (1) as diagnostic biomarkers for early NAFLD, where genes correlated with steatosis severity could enable non-invasive screening via blood or tissue expression profiles; and (2) as therapeutic targets in MDD, where dysregulated immune-metabolic pathways may guide personalized pharmacotherapy. Furthermore, our transcriptomic-focused analysis would benefit from integrating proteomic/metabolomic data to elucidate mechanistic pathways. We plan to address these gaps by expanding independent cohorts and validating key genes through immunohistochemistry and functional assays.

## Conclusion

In summary, despite these limitations, our study has made significant strides in advancing the early diagnosis of NAFLD in individuals with MDD. By developing a robust machine-learning model anchored in an eight-gene signature, we provide a promising clinical tool to facilitate the early identification and management of NAFLD. Future research should prioritize validation in larger, more diverse cohorts and the discovery of additional biomarkers to enhance model predictive power. Ultimately, this work paves the way for precision medicine approaches tailored to manage this complex comorbidity.

## Data Availability

The original contributions presented in the study are included in the article/Supplementary Material, further inquiries can be directed to the corresponding authors.
